# Frequency, types and predictors of drug therapy problems among non-dialysis chronic kidney disease patients at a tertiary care hospital in Pakistan

**DOI:** 10.1371/journal.pone.0284439

**Published:** 2023-04-14

**Authors:** Muhammad Hayat, Nafees Ahmad, Syed Mohkumuddin, Syed Liaquat Ali Khan, Amer Hayat Khan, Noman Ul Haq, Naheed Haque, Amjad Khan

**Affiliations:** 1 Department of Pharmacy Practice, Faculty of Pharmacy and Health Sciences, University of Balochistan, Quetta, Pakistan; 2 Department of Nephrology, Bolan Medical College, Quetta, Pakistan; 3 Discipline of Clinical Pharmacy, School of Pharmaceutical Sciences, Universiti Sains Malaysia, Pulau Pinang, Malaysia; 4 Sardar Bahadur Khan Women’s University, Quetta, Pakistan; 5 Department of Pharmacy, Quaid-i-Azam University, Islamabad, Pakistan; Osaka University of Pharmaceutical Sciences, JAPAN

## Abstract

**Background:**

Drug therapy problems (DTPs) are common among patients suffering from chronic kidney disease (CKD). However, there is a lack of information about DTPs and its predictors among CKD patients from Pakistan.

**Objectives:**

To evaluate the frequency, type and predictors of various types of DTPs among CKD patients at a tertiary-care hospital in Pakistan.

**Methodology:**

This was a cross-sectional study carried out at Sandeman Provincial Hospital, Quetta between 1-11-2020 and 31-1-2021. It included 303 non-dialysis ambulatory patients of CKD-stage 3 and above. Cipolle et al., criterion was used for classifying the DTPs and a clinician at the study site checked the identified DTPs for accuracy. Data were analyzed by SPSS 23. Multivariate analysis was conducted to find the predictors of individual types of DTPs. A p-value <0.05 was considered statistically significant.

**Results:**

The patients received a total of 2265 drugs with a median of eight drugs per patient (range: 3–15 drugs). A total of 576 DTPs were identified among 86.1% patients with a median of two DTPs (interquartile range 1–3) per patient. *Dosage too high* (53.5%) was the most common DTP followed by *adverse drug reactions (ADRs)* (50.5%) and *need of additional drug therapy* (37.6%). In multivariate analysis, patients’ age of >40 years emerged as a predictor of *unnecessary drug therapy* and *dosage too high*. The odds of *needing a different drug product* was significantly high in patients with cardiovascular diseases (CVD) and diabetes mellitus (DM). The *dosage too low* had significant association with CVD. The risk of *ADRs* was significantly high in elderly patients (>60 years) and those with CVD. The presence of hypertension, DM and CKD stage-5 emerged as predictors of *dosage too high*.

**Conclusion:**

This study revealed a high prevalence of DTPs among CKD patients. Targeted interventions in high risk patients may reduce the frequency of DTPs at the study site.

## Introduction

Chronic kidney disease (CKD) defined as “kidney damage or glomerular filtration rate (GFR) <60 ml/min/1.73 m^2^ for three months or more, irrespective of cause” is a global public health problem [1]. In 2017, it was estimated that 9.1% of the world population had CKD and 1.2 million deaths were attributed to it [1]. It is important to mention that the significance of CKD lies not only in the burden of adverse consequences associated with the disease itself but also in the burden associated with the use of drugs to manage it [2]. Chronic kidney diseases are associated with many complications like hypertension, anemia, diabetes mellitus (DM), cardiovascular diseases (CVD), dyslipidemia, mineral bone disease etc. Therefore, CKD patients treated with several heterogeneous medications to halt the progression of CKD and minimize complications [3–5]. The impaired renal function, presence of co-morbidities, polypharmacy, dose adjustment needed for various drugs and older age make CKD patients a high risk group for occurrence of drug therapy problems (DTPs) [2, 6, 7]. A DTP is a gap between the prescribed therapy and the one needed to achieve the desired therapeutic outcomes [8]. Examples of DTPs include *unnecessary drug therapy*, *need of additional drug therapy*, *need of a different drug product*, *dosage too low*, *adverse drug reaction*, *dosage too high* and *non-adherence* [2, 8]. The presence of DTPs not only interferes with the desired therapeutic outcomes, but also results in increased morbidity, hospitalizations, mortality, and health care costs [5, 7, 9, 10]. In the published literature, the prevalence of DTP among CKD patients ranges from 55.9–100% [2, 11–16]. The degree of renal impairment, patients’ age and number of prescribed medicines and comorbidities are the commonly reported predictors of DTPs among CKD patients [2, 11–15]. The overview of prevalence, categories and predictors of DTPs among CKD patients in studies conducted in different countries around the world are presented in Table 1.

**Table 1:** Prevalence, categories and predictors of DTPs among CKD patients.

Study	Country	Prevalence of DTPs (%)	Most common categories of DTP	Predictors
[2]	Kenya	100	Drug-drug interaction, indication without drug	CKD stage 4, number of medications per prescription and number of comorbidities per patient
[11]	France	93	Indication without drug, low dose, need of different drug	Number of drugs prescribed and patients’ age
[12]	Norway	62	Incorrect dose, need of different drug	Deteriorating renal function and number of drugs prescribed
[13]	Ethiopia	78.6	Need of additional drug, non-adherence, low dose	Polypharmacy, number of comorbidities and CKD stage
[14]	India	76.6	Non-adherence, low dose, need of additional drug	Presence of comorbidities
[15]	Jordan	99.2	Efficacy related problems, indication related problems, low dose	Not evaluated
[16]	Ethiopia	55.9	Need of additional drug, non-compliance, unnecessary drug therapy, low dose	Patients’ occupation and use of angiotensin converting enzymes inhibitor

CKD, chronic kidney diseases; DTPs, drug therapy problems

Pakistan with a population >200 million is a CKD high burden country. Alarmingly, the findings of a systematic review revealed that >21.2% of Pakistanis suffer from CKD [17, 18]. Getting information about the frequency, types and predictors of various types of DTPs could help in identifying the high risk groups and designing the targeted interventions to improve the clinical practice and reduce the morbidity, mortality and healthcare costs associated with DTPs [5, 19, 20]. Despite harboring a high burden of CKD patients, there is a scarcity of published information regarding DTPs among CKD patients from Pakistan. Therefore, the current study is conducted to evaluate the prevalence, types and predictors of various types of DTPs among CKD patients treated at a tertiary care hospital in Pakistan.

## Materials and methods

### Study settings and design

This was a cross-sectional analytical study carried out at nephrology department of Sandeman Provincial Hospital (SPH) Quetta, Balochistan. The study site is an 800 bedded, largest tertiary care hospital of the province with a wide catchment area of the whole province and border areas of nearby Afghanistan and Iran. Approximately 8000–10000 patients daily visit the outdoor patient departments (OPD) of SPH for different diseases [21, 22].

### Study patients

All established non-dialysis CKD (ND-CKD) patients who were ≥18 years old, visited the nephrology OPD of SPH between November 1, 2020 and January 31, 2021 and willing to participate in the study by giving oral and written consent (in case of educated patients) were included in the study. The eligible patients were identified by reviewing their medical charts. Their glomerular filtration rate (GFR) was calculated by using CKD-Epi formula [23] and they were categorized into CKD stage 3a (GFR = 45–59 ml/min/1.73 m^2^), stage 3b (GFR = 30–44 ml/min/1.73 m^2^), stage 4 (15–29 ml/min/1.73 m^2^) and stage 5 (<15 ml/min/1.73 m^2^) [24]. Those patients who had GFR ≥ 60 ml/min/1.73 m^2^, were pregnant or had renal transplant were excluded from the study. Sample size was calculated by using Daniel’s sample size calculation formula, i.e., Z^2^P (1−P)/d^2^ [25], where n = required sample size, Z = Z-statistics for a level of confidence (for 95% level of confidence, Z = 1.96), P = expected prevalence in population based on previous published studies [in proportion of 1, so, the estimated frequency of DTPs among CKD patients was 76.6% or 0.766 [14]], d = absolute error or precision (in proportion of 1, if 5%, d = 0.05). By putting these values in Daniel sample size calculation formula, the minimum number of patients required for this study was to 275.

### Data collection and identification of DTPs

Patients’ data were collected through a standardized data collection form devised on the basis of extensive literature review and suggestions from supervisory committee and clinical team at the study site. The collected data included patients’ age, gender, comorbidity, stages of CKD, results of laboratory tests, the medications prescribed and DTPs. Diagnosis of comorbidities was based on documentation in the patients’ medical record [26–28]. Drugs prescribed were noted by their generic names and classified under their respective pharmacological classes [26–28]. Cipolle et al., criterion was used for identification and classification of DTPs [8]. The said criterion classifies the DTP into seven distinct categories i.e., i) *unnecessary drug therapy* ii) *needs additional drug therapy* iii) *needs different drug product* iv) *dosage too low* v) *adverse drug reaction* vi) *dosage too high and* vii) *non-adherence* [8]. However, as the majority study participants were uneducated, therefore it was not possible to evaluate the subjective medication adherence by a self-administered tool. Therefore, we evaluated only the first six categories of DTPs. In order to ensure the appropriateness of medications prescribed, their doses, duration, compelling indications for prescription and beneficial drug-drug interactions (DDIs), a detailed review of the patients’ medical record was conducted. The DTPs were identified by the principal investigator and checked for accuracy by a clinician at the study site. Drug-drug interactions were checked by Lexicomp interact^®^ [29], a highly reliable and valid software used for identifying DDI. On the basis of level of urgency and timely response towards these DDIs, Lexicomp interact^®^ classifies DDIs into five categories i.e. A (no known interaction), B (no action needed), C (monitor therapy: it is documented that the benefits of an interaction outweigh the risks, in order to avoid potential adverse outcomes, closely monitor the therapy), D (consider therapy modification: it is documented that proper actions must be taken to reduce the toxicity resulting from this interaction), X (avoid combination: the risk of an interaction outweighs the benefits and are usually contraindicated) [22, 29]. In order to avoid the inclusion of beneficial DDIs as a DTP, we included only D and X DDIs in the current study.

### Statistical analysis

Data were analyzed using Statistical Package for Social Sciences (SPSS version 23). All categorical data were presented as frequencies and proportions, whereas means along with standard deviations and medians along ranges were used for displaying continuous data. Multivariate binary logistic regression (MVBLR) analysis was conducted to find the predictors of individual types of DTP. The inclusion of independent variables in univariate analysis were based on literature review, suggestions from the supervisory team and clinical relevance of these factors with DTPs among CKD patients in the published studies [2, 11–15, 30]. All those variables which had association at a p-value <0.2 with individual types of DTP in univariate analysis were entered into MVBLR analysis after checking for collinearity. If two variables had high collinearity (tolerance value <0.1 and/or variance inflation factor >10) one of them was excluded. A p-value <0.05 was considered statistically significant.

### Ethics approval

This study was approved by the Research and Ethics Committee of Faculty of Pharmacy and Health Sciences, University of Balochistan, Quetta, Pakistan. All eligible patients with who were willing to participate in the study gave oral or written consent (in case of educated patients or their treatment supporters).

## Results

### Socio-demographic and clinical characteristics of patients

A total of 303 ND-CKD patients were included in the current study. The median age of patients was 55 years (interquartile range from 45–60 years). Majority of patients were males (56.4%), had an age of 41–60 years (55.4%), body weight of >80 kg (55.4%), suffered from CKD stage 5 (40.6%) and had a comorbidity (98.7%) with hypertension being the most common one (84.2%) followed by anemia (78.2%). Cross-tabulation between patients’ characteristics and CKD stages are presented in Table 2.

**Table 2:** Cross-tabulation between patients’ characteristics and CKD stages.

Variable	No (%)	CKD stage No. (%)			p-value
		3	4	5	
**Gender**					<0.001
Male	171 (56.4)	57 (33.3)	42 (24.6)	72 (42.1)	
Female	132 (43.6)	18 (13.6)	63 (47.7)	51 (38.6)	
**Age** (years)					<0.001
≤40	63 (20.8)	33 (52.4)	15 (23.8)	15 (23.8)	
41–60	168 (55.4)	42 (24.4)	53 (31.5)	74 (44.0)	
>60	72 (23.8)	1 (1.4)	37 (51.4)	34 (47.2)	
**Weight** (kilogram)					<0.001
41–60	27 (8.9)	15 (55.6)	6 (22.2)	6 (22.2)	
61–80	108 (35.6)	27 (25.0)	30 (27.8)	51 (47.2)	
>80	168 (55.4)	33 (19.6)	69 (41.1)	66 (39.3)	
**Presence of a comorbidity**	299 (98.7)	74 (24.7)	102 (34.1)	123 (41.1)	0.129*
**Number of comorbidities**					<0.001*
0	4 (1.3)	1 (25.0)	3 (75.0)	0 (0.0)	
1	65 (21.5)	35 (53.8)	18 (27.7)	12 (18.5)	
2	99 (32.7)	24 (24.2)	36 (34.6)	39 (39.4)	
3	120 (39.6)	12 (10.0)	48 (40.0)	60 (50.0)	
4	15 (5.0)	3 (20.)	0 (0.0)	12 (80.0)	
**Hypertension**	255 (84.2)	63 (24.7)	93 (36.5)	99 (38.8)	0.249
**Anemia**	237 (78.2)	39 (16.5)	81 (34.2)	117 (49.4)	<0.001
**DM**	132 (43.6)	24 (18.2)	45 (34.1)	63 (47.7)	0.030
**CVD**	78 (25.7)	9 (11.5)	21 (26.9)	48 (61.5)	<0.001
**Others**	15 (5.0)	3 (20.0)	9 (60.0)	3 (20.0)	0.094

CKD, chronic kidney diseases; CVD, cardiovascular diseases; DM, diabetes mellitus

*Fisher exact test

### Drugs prescription pattern

The study participants received a total 2265 drugs with a median of eight drugs per patient (range: 3–15 drugs). The most commonly prescribed medications were anti-acidotic agents (n = 267; 88.1%), followed by antihypertensive (n = 252, 83.2%) and vitamin D preparations (n = 215, 70.9%). Therapeutic classes of drugs and cross-tabulation between drugs prescribed and CKD stages are given in Table 3.

**Table 3:** Drugs prescription pattern.

Drugs prescribed	No. (%)	CKD stage No. (%)			p-value
		3	4	5	
**Anti-acidotic agents**	267 (88.1)	60 (60.0)	90 (85.7)	117 (91.8)	0.004
**Anti-hypertensives**	252 (83.2)	61 (24.2)	78 (31.0)	113 (44.8)	<0.001
**RASi**	123 (40.6)	40 (32.5)	26 (21.1)	57 (46.3)	0.001
**CCBs**	165 (54.5)	38 (23.0)	62 (37.6)	65 (39.4)	0.483
**Beta-blockers**	72 (23.8)	3 (8.3)	15 (20.8)	54 (70.8)	<0.001
**Diuretics**	135 (44.6)	24 (17.8)	33 (24.4)	78 (57.9)	<0.001
**Alpha blockers**	15 (5.0)	5 (33.3)	2 (13.3)	8 (53.3)	0.205
**Anti-anemics**	168 (55.4)	24 (14.3)	63 (37.5)	81 (48.2)	<0.001
**Anti-emetics**	165 (54.5)	35 (21.2)	53 (32.1)	77 (46.7)	0.055
**Vitamin-D preparations**	215 (71.0)	41 (19.1)	70 (32.6)	104 (48.4)	<0.001
**Anti-diabetics**	109 (36.0)	16 (14.7)	42 (38.5)	51 (46.8)	0.009
**Biguanides**	33 (10.9)	6 (18.2)	21 (63.6)	6 (18.2)	0.001
**Sulfonylureas**	42 (13.9)	4 (9.5)	14 (13.3)	24 (57.1)	0.019
**Gliptins**	54 (17.8)	6 (11.1)	20 (37.0)	28 (51.9)	0.029
**NSAIDs**	91 (30.0)	27 (29.7)	42 (46.2)	22 (24.2)	<0.001
**Statins**	65 (21.5)	10 (15.4)	16 (24.6)	39 (60.0)	0.001
**PPIs**	85 (28.1)	12 (14.1)	28 (32.9)	45 (52.9)	0.007
**Anti-platelets**	77 (25.4)	10 (13.0)	24 (31.2)	43 (55.8)	0.002
**Antibiotics**	24 (7.9)	14 (58.3)	7 (29.2)	3 (12.5)	<0.001
**Antipsychotics**	42 (13.9)	9 (21.4)	26 (61.9)	7 (16.7)	<0.001
**Multi-vitamins**	27 (8.9)	7 (25.9)	11 (40.7)	9 (33.3)	0.698

CKD, chronic kidney disease; CCBs, calcium channel blockers; NSAIDs, non-steroidal anti-inflammatory drugs; PPIs, proton pump inhibitors; RASi, renin angiotensin system inhibitors

### Frequency, types and predictors of drug therapy problems

In the current study, a total of 576 DTPs were identified. Majority of patients (86.1%, n = 261) had at-least one DTP with a median two DTPs (interquartile range 1–3) per patient. Of 261 patients with DTPs, 93 had one, 87 two and 81 had three or more DTPs. *Dosage too high* (n = 162, 53.5%) was the most common class of DTP, followed by *ADRs* (n = 153, 50.5%) and *needs additional drug therapy* (n = 114, 37.6%) (Table 4).

**Table 4:** Types of DTPs.

Drug therapy problems category	Types of drug therapy problems	No. (%)
**Unnecessary drug therapy**	**Yes**	**42 (13.9)**
	No medical indication	18 (5.9)
	Duplicate therapy	21 (6.9)
	No medical indication + duplicate therapy	3 (1.0)
**Needs additional drug therapy**	**Yes**	**114 (37.6)**
	Untreated indication	96 (31.7)
	Synergistic / potentiation	9 (3.0)
	Untreated indication + synergistic	9 (3.0)
**Needs different drug product**	**Yes**	**81 (26.7)**
	More effective drug available	69 (22.8)
	Dosage forms inappropriate	3 (1.0)
	Not effective for condition	9 (3.0)
**Dosage too low**	**Yes**	**21 (6.9)**
	Wrong dose	3 (1.0)
	Drug interaction	18 (5.9)
**Adverse drug reaction**	**Yes**	**153 (50.5)**
	Drug interaction	105 (34.7)
	Contraindication	48 (15.8)
**Dosage too high**	**Yes**	**162 (53.5)**
	Wrong dose	96 (31.7)
	Frequency inappropriate	60 (19.8)
	Drug interaction	9 (3.0)

### Predictors of DTPs

The results of multivariate analysis (Table 5) revealed that various demographic and clinical characteristics had statistically significant association with individual types of DTPs. The presence of *unnecessary drug therapy* was significantly high in patients of age >40 years. Presence of anemia and CVD had statistically significant positive association with *need for additional drug therapy*. Presence of DM and CVD emerged as predictors for *needing a different drug product*. Presence of CVD was significantly associated with *dosage too low*. The odds of *ADRs* was significantly high in those of age >60 years and who suffered from CVD. Whereas, patients’ age of >40 years, CKD stage 5, and presence of hypertension and DM emerged as predictors of *dosage too high*.

**Table 5:** Predictors of various types of DTP.

Type of DTP	Predictor	No. (%)	Univariate analysis OR (95% CI)	p-value	Multivariate analysis OR (95% CI)	p-value
Unnecessary DTP	41–60 Age (years)	30 (17.9)	4.255 (1.250–14.481)	0.020	4.938 (1.402–17.385)	0.013
	>60 Age (years)	9 (12.5)	3.000 (0.774–11.621)	0.112	4.351 (1.010–18.737)	0.048
	CKD stage 4	12 (11.42)	0.592(0.296–1.236)	0.151	0.457(0.231–1.321)	0.070
Needs additional drug therapy	weight>80 Kg	55 (32.73)	0.645 (0.338–1.346)	0.160	0.460 (0.232–1.092)	0.060
	Anemia	105 (44.3)	5.038 (2.384–10.647)	<0.001	6.758 (2.895–15.775)	<0.001
	CVD	48 (61.5)	3.855 (2.249–6.607)	<0.001	3.964 (2.174–7.228)	<0.001
Needs different drug product	DM	54 (40.9)	3.692 (2.156–6.323)	<0.001	5.385 (2.643–10.970)	<0.001
	CVD	30 (38.5)	2.132 (1.227–3.706)	0.007	3.331 (1.654–6.707)	0.001
Dosage too low	CVD	9 (11.5)	2.315 (0.936–5.728)	0.069	3.562 (1.298–9.772)	0.014
Adverse drug reaction	>60 Age (years)	45 (62.5)	4.687 (2.242–9.802)	<0.001	2.688 (1.116–6.476)	0.028
	DM	78 (59.1)	1.849 (1.167–2.929)	0.009	1.572 (0.921–2.684)	0.097
	CVD	57 (73.1)	3.647 (2.071–6.422)	0.000	4.287 (2.244–8.191)	<0.001
Dosage too high	41–60 Age (years)	105 (62.5)	9.545 (4.420–20.614)	<0.001	4.127 (1.660–10.261)	0.002
	>60 Age (years)	48 (66.7)	13.714 (5.732–32.810)	<0.001	4.193 (1.367–12.861)	0.012
	Hypertension	144 (56.5)	2.162 (1.146–4.079)	0.017	2.680 (1.167–6.155)	0.020
	DM	99 (75.0)	5.143 (3.114–8.494)	<0.001	4.374 (2.255–8.481)	<0.001
	CVD	54 (69.2)	2.437 (1.410–4.214)	0.001	1.968 (0.952–4.069)	0.068
	CKD stage 5	99 (80.5)	13.062 (6.535–26.110)	<0.001	11.013 (4.355–27.851)	<0.001

CI, confidence interval; CKD, chronic kidney disease; CVD, cardiovascular disease; DM, diabetes mellitus; OR, odds ratio

* Only those variables are presented in the table which were included in multivariate analysis

## Discussion

This study has evaluated the frequency, pattern, and predictors of various types of DTPs among ambulatory ND-CKD patients of stage 3 and above at a tertiary care hospital in Pakistan. A total of 86.1% patients had at-least one DTP with *dosage too high* (53.5%) being most common class of DTPs followed by *ADRs* (50.5%) and *need of additional drug therapy* (37.6%). Frequency of DTPs in this study was in the range (62–100%) reported by studies conducted elsewhere [2, 11–15]. The variation in prevalence of DTPs among CKD patients reported by published studies could be due to differences in study populations, the tools and methods used for detecting and classifying DTPs, the study designs and duration, prescribing practices and availability of clinical pharmacists at the study sites [2, 11–16].

In this study, the prevalence of *dosage too high* (53.5%) was comparatively higher than the range reported in relevant published literature (15–42.2%) [7, 11, 31]. This could be due to the high number CKD stage 4 and 5 patients (75.3%) in current cohort. The decreased renal function warrants dose reduction for certain drugs. For instance, among CKD patients with eGFR<30 ml/min/m^2^, certain antihypertensive (captopril, enalapril, esinopril, rimipril, atenolol, bisoprolol, hydrcholorothiazide, spironolocatone, amililoride), antidiabetic (metformin, SGLT2i inhibitors) and lipid lowering drugs (fluvastatin, rosuvastatin) need dose reduction. However, it has been noted that doctors find it challenging to do the recommended dose adjustment in patients with advanced CKD [32, 33]. This observation is supported by the emergence of CKD stage 5 and presence of hypertension and DM as predictors of *dosage too high* in this study. In the current cohort, *ADRs* were relatively more prevalent (50.5%) than its range (8.9–45%) reported elsewhere [9, 34, 35]. In this study, DDIs and contraindications to the use of particular drugs respectively made 68.6% and 31.4% of ADRs, while patients’ age >60 years and suffering from CVD emerged as predictors of *ADRs*. Elderly patients and those sufferings from CVD are usually treated with multiple heterogeneous drugs. Therefore, these patients are at greater risk of DDIs [22, 36]. In the present study, clopidogrel + omeprazole, amlodipine + calcium acetate and carvedilol + nifedipine were the common clinically significant interacting pairs, while NSAIDs, metformin and hydrochlorothiazide were the contraindicated drugs prescribed to CKD stage-5 patients [18, 29, 37, 38]. We found that the *need of additional drug therapy* was significantly high among anemic and CVD patients. As the presence of comorbidities complicates the management of CKD, and consequently increases the likelihood of doctors missing out on the diagnosis and treatment of some comorbidities, and failure to potentiate the therapy in those who are not at desired goals. Anemia is a common comorbidity and important component of care among CKD patients [39–42]. It is associated with decreased quality of life [43] and increased morbidity and mortality [44]. The suboptimal management of anemia among CKD patients in this study is in compliance with the previous reports from elsewhere [43–45]. The doctors’ irrational practices, existing controversies regarding anemia management among CKD patients in terms of hemoglobin levels for treatment initiation, the medication choice and target biochemical levels to avoid potential adverse events could be some of the potential reasons for suboptimal management of anemia in these patients [39]. Furthermore, the use of erythropoietin stimulating agents in CKD patients with hemoglobin level >11 gm/dl has also been associated with increased cardiovascular events [39]. In addition to be a common comorbidity, CVD is the leading cause of mortality and morbidity among CKD patients [39, 46]. In compliance with our finding, the suboptimal prescription of key cardiovascular medicines (ACEi/ARBs, antiplatelets and statins) among CKD patients suffering from CVD has previously been reported by studies conducted elsewhere [47, 48]. The fear of adverse events (i.e. hyperkalaemia, worsening of kidney function) caused by these drugs in advanced CKD (eGFR <30 ml/min/m^2^) could be a possible reason for suboptimal prescription of these agents [49]. However, in the absence of absolute contraindications in less severe CKD stages, the suboptimal prescription of these drugs would negatively impact the patients’ clinical outcomes [47, 48]. We observed that the odds of *dosage too low* were significantly high among CVD patients. On further analysis, we found that 18/21 DTPs of *dosage too low* were due pDDIs (Table 4) and the prescription of interacting pair of clopidogrel (pro-drug and substrate of CYP2C19) + omeprazole (inhibitor of CYP2C19) [22, 50] to CVD patients made them a high risk group for the DTP of *dosage too low*.

It is important to mention that results of this single center study should be interpreted with some noteworthy limitations of a cross-sectional design and convenient enrollment of study participants. As majority of patients were uneducated, it was not possible to evaluate their subjective medication adherence by a self-administered tool. Due to lack of information about patients’ height we were also not able to measure their body mass index. Furthermore, this study did not evaluate the impact of presence DTPs on patients’ clinical outcomes. A multicenter, large prospective study with random patients’ selection and evaluating the impact of DTPs on patients’ clinical outcome is suggested to confirm findings of the current study.

## Conclusion

We observed a high prevalence of DTPs among ND-CKD patients treated at a tertiary care hospital in Pakistan. In order to reduce the incidence of DTPs among CKD patients, multifaceted strategies should be adopted. Pharmacists, due to their broad knowledge on drug use are best positioned to ensure the rational use of medicines by identifying, preventing and resolving DTPs. Incorporating clinical pharmacists into multidisciplinary teams treating CKD patients could reduce the incidence of DTPs and improve patient outcomes. Furthermore, the availability of DDIs screening tools in healthcare settings, dissemination and implementation of CKD management guidelines, clinical decision support software, doctors’ alert systems, and the development and implementation of precautionary guidelines could help in reducing the incidence of DTPs among CKD patients at the study site and other hospitals in Pakistan.
